# Cypripedium in Amenorrhœa and Dysmenorrhœa

**Published:** 1876-12

**Authors:** G. H. Batte

**Affiliations:** Virginia


### CYPRIPEDIUM IN AMENORRHŒA AND DYSMENORRHŒA.

BY G. H. BATTE, M. D., OF VIRGINIA.

For a further illustration and recommendation of the virtues of cypripedium in the treatment of cases of amenorrhœa and dysmenorrhœa, which was the subject of my last communication, I beg leave to report the following cases which have fallen under my care:

CASE I.—Miss W., aged 18, had never menstruated but twice at the age of 16, with scanty flow. On presentation she mani fested the following symptoms: Periodical headache, with vertigo; pain in sacral region; œdema of lower extremities, and tympanitic abdomen. She had been treated with iron and aloes and various domestic remedies without relief—never having any show beyond a slight leucorrhœa. For six weeks continuously I gave her the tr. of cypripedium three times daily, beginning with one drachm and gradually increasing the dose, with the effect of establishing a regular and natural discharge, affording entire relief to all her complaints. Since then she has married, and given birth to twins.

Case II.—Miss F., of Vermont, aged 18, had menstruated regularly since her 14th year, but with scanty flow, accompanied with ever-recurring pelvic and lumbar pains and headache. She removed from Vermont to Virginia, hoping that a change of climate would be beneficial. After remaining here for six months, without relief, she applied to me for treatment. I put her immediately on the fluid extract cypripedium as prepared by Dr. G. C. Stark, of Petersburg, Va., giving her a drachm three times daily, increasing it to six drachms daily during the period. She had only taken the extract for one week, when the menstrual flow appeared of natural quantity with entire absence of pain. The relief thus afforded continued for eighteen months, when she again applied to me, stating that her pains had returned. The fluid extract cypripedium again afforded her immediate relief.

Case III.—Miss G., from Vermout, aged 17, had menstruated regularly since her 16th year, with scanty flow, continuing five days at each period, accompanied with excruciating pain in the back and pelvis, marked with great severity on the first, second and third days. She had been under treatment of physicians in her native State, who, she said, had given her quinine, camphor and belladonna, without affording permanent relief. The fluid extract cypripedium, given as in the foregoing case, cured her pains and re-established a full and free discharge.

My principal design in publishing the above cases, is to show the mode of administering the remedy, and the principal conditions which indicate its use. Some physicians of standing have prescribed it in menorrhagia, but I cannot think it applicable here. Even in cases of amenorrhœa and dysmenorrhœa, where there is mechanical obstacles to the flow by stricture or displacement, of course these must be removed by proper appliances. I have frequently noticed that curvature of the neck is a fruitful source of dysmenorrhœa, and requires long patience to cure it. I always give the cypripedium in conjunction with other treatment.

